# Microorganism's adaptation of Crucian carp may closely relate to its living environments

**DOI:** 10.1002/mbo3.650

**Published:** 2018-06-06

**Authors:** Huanxin Zhang, Hongshuo Tang, Yu Zang, Xuexi Tang, Ying Wang

**Affiliations:** ^1^ College of Marine Life Sciences Ocean University of China Qingdao China; ^2^ Laboratory of Marine Ecology and Environmental Science Qingdao National Laboratory for Marine Science and Technology Qingdao China; ^3^ College of Information Science and Engineering Ocean University of China Qingdao China

**Keywords:** 16S rRNA, adaptation, Crucian carp, internal microbiome

## Abstract

The relationship between the internal microbiome of an individual organism and that of its external environment has been little investigated in freshwater ecosystems. Thus, this is an area of interest in freshwater fish biology. Along with the genotype of the fish host, external environment plays an important role in determining the composition of the internal microbiome. Here, we characterized the variability of the microbiome of wild Crucian carp (*Carassius auratus*), along with those of their surrounding environments (water and mud). We found that each environment had distinct bacterial communities, with varying composition and structure. The primary bacterial phyla identified in the Crucian carp gut were Fusobacteria and Proteobacteria (90% of all bacterial phyla identified); the primary genera identified were *Cetobacterium*,* Aeromonas*, and *Plesiomonas* (85% of all bacterial phyla identified). We identified 1,739 operational taxonomic units (OTUs) in the Crucian carp gut, 1,703 in water, and 5,322 in mud. Each environment had unique OTUs, but the fewest unique OTUs (97) were found in the Crucian carp gut. There were significant differences in the relative abundances of different bacterial phyla in the different environments. It may be that only bacterial phyla vital for efficient fish function (e.g., immune response or metabolism), such as Fusobacteria and Proteobacteria, are retained in the Crucian carp gut.

## 1 INTRODUCTION

Associations between the intestinal microecology of individual organisms and the environment have been frequently studied in recent years (Beckers, Op, Weyens, Boerjan, & Vangronsveld, 2017; Hu et al., 2017). This keen interest is reflected by numerous large projects, including the National Microbiome Initiative (Gordon et al., 2005) and human microbiome project (Turnbaugh et al., 2012). Projects have ranged in scale from the gut microbiomes of individual organisms to the microbiota associated with all living organisms (Chen et al., 2017; Glenwright et al., 2017; Wu et al., 2017).

It is clear that intestinal microecology is closely related to the external environment for reasons that are, in most cases, linked to nutrient acquisition, and are therefore crucial to the performance and survival of the host organism. Intestinal bacterial communities may also play important roles in the regulation of the body's immune system (Chan et al., 2016; Fukuda & Ohno, 2014; Tun et al., 2014; Wei, Wang, & Wu, 2015; Wu et al., 2016). Indeed, microbiomes have been called the host's second or extended genome (Zhang et al., 1873).

Fish are common in aquatic ecosystems. The interactions between the intestinal microbiomes of fish and the external environment are of great interest. In addition, the fish intestinal microbiome is one of the key determinants of fish health: bacterial microbiota may improve nutrient bioavailability and extraction from water or mud, as well as increase host tolerance of and resistance to biotic (and abiotic) stressors (Beckers et al., 2017). In exchange, the host fish provides a safe habitat and a constant supply of energy to the microbiota (Gill et al., 2006). Bacterial communities exist in virtually all global environments; these microenvironments provide specific biotic and abiotic conditions for the residing bacterial communities (Fierer & Jackson, 2006).

Within microbiome research, most attention has been directed toward the bacterial communities inhabiting the fish farm environment or within the fish intestine (Indugu, Bittinger, Kumar, Vecchiarelli, & Pitta, 2016; Sun et al., 2016; Wu et al., 2016, 2017; Zhuang et al., 2016). For example, Stephens et al. (2016) observed that microbial community composition varied with zebrafish age and developmental stage. While, Harnisz M et al. reported that a freshwater fish farm impacted the tetracycline‐resistant bacterial community, as well as the structure of tetracycline‐resistant genes in river water.

Although several studies are available which treat the bacterial communities of the fish intestine and the fish farm separately, few studies have focused on the interactions between the fish intestinal microbiomes of fish (such as Crucian Asia) and their surrounding fish farm environment.

Crucian carp (*Carassius auratus*) are omnivorous, freshwater fish common in Central Asia and China (Brönmark & Miner, 1992). These fish are popular with consumers as their meat is tender, high in protein, tasty, and contains nutrients such as calcium, phosphorus, and iron. Furthermore, Crucian carp are easily cultivated (Pettersson, Andersson, & Nilsson, 2001). The publication of the Crucian carp genome, along with the availability of detailed historical breeding pedigrees and newly developed gene editing technologies, may allow the establishment of accelerated breeding programs and genetic engineering projects in this species.

Crucian carp have quickly come to dominate freshwater fisheries. There is a strong correlation between the internal microbiota and organism health (e.g., immune response and metabolism; Lv et al., 2016; Zhuang et al., 2016). It is therefore important to compare the microbiota of the Crucian carp gut to the microbiota of its external environment.

Here, we aimed to compare the niche differentiation of the microbiota associated with the Crucian carp gut, to that of the microbiota associated with water and mud using high‐throughput sequencing of two of the hypervariable regions (V3 and V4) of the 16S rRNA gene. We also aimed to investigate the relationships among these three microbiotas.

## 2 MATERIALS AND METHODS

### 2.1 Sample collection

In November 2016, we established about one and a half acres enclosure in a freshwater lake located in Jining, Shandong, China (34°29′N, 116°36′E), and populated it with Crucian carp at a density of 200 fish per acre. Original species and an enclosure can ensure the original connection between Crucian carp and environments. Crucian carp were maintained in this enclosure for 5 months without anti‐inflammatory drugs or antimicrobials. In April 2017, we collected eight fish (JY: JY1–8; average weight: approximately 0.6 kg) at random using a self‐made device. This self‐made device included a net, an organic glass hydrophore, and a bottom sampler. Fish were placed in sterile tubes and were immediately killed by freezing at −80°C. Intestinal contents were removed for DNA extraction.

Also in April 2017, we collected 10 water samples (ST: ST1–ST10) and nine samples of mud (YN: YN1–YN9). Water and mud samples were collected from the same location as the fish. Water samples were collected at a depth of approximately 5 m; mud samples were collected 5–10 cm below ground level. Water and mud samples were immediately placed into sterile plastic tubes, frozen in the field, and stored in our laboratory at −80°C.

All procedures performed on animals were conducted in accordance with the ethical standards of the Qufu Normal University Animal Care and Use Committee (Permit Number: QFNU2015‐002).

### 2.2 DNA extraction, PCR amplification, and 16S rRNA sequencing

DNA was extracted in duplicate from the intestinal content, water, and mud samples. To that our intestinal samples were comprehensive and representative, individual Crucian carp intestinal samples were mixed. For each DNA extraction, we used approximately 250 mg of intestinal contents, 10 L of water, or 250 mg of mud. DNA was extracted using a QIAamp DNA Kit (Qiagen, Germany), following the manufacturer's protocol.

We amplified the V3–V4 region of the 16S rRNA bacterial gene using specific primers carrying the Illumina MiSeq sequencing adapter (16S Amplicon PCR forward: 5′‐CTACGGGNGGCWGCAG‐3′ and 16S Amplicon PCR reverse: 5′‐GACTACHVGGGTATCTAATCC‐3′, Wu et al., 2016). We prepared the PCR mix using the KAPA HiFi Hot Start Ready Mix (2×) (TaKaRa Bio Inc., Japan), following the manufacturer's instructions. We used the MiSeq PCR conditions described in Wu et al. (2016), and purified the 16S rRNA amplicons with AMPure XP beads (Beckman, USA), following the manufacturer's instructions. Extracted DNA from each of the 10 samples of each microbiota was pooled in equal concentrations prior to sequencing on an Illumina MiSeq platform (Illumina MiSeq sequencing system, USA).

### 2.3 Statistical analysis

Raw sequencing reads were demultiplexed and quality filtered using mothur v1.39.5. When processing raw sequencing reads, there is always a possibility that nontarget sequences may be coamplified. First, bacterial 16S rRNA is highly homologous to other 16S sequences, including chloroplast 16S rRNA and mitochondrial 16S rRNA. Second, chimeras (hybrid sequences) are common when amplifying closely related sequences. Both of these errors may artificially inflate diversity estimates. We therefore conservatively removed suspect sequences based on existing databases. Both of these problems can artificially inflate diversity estimates. We determined the operational taxonomic units (OTUs) comprising the microbiomes with Uparse v7.0.1001 (http://drive5.com/uparse), where OTUs were defined has having at least 97% sequence similarity. We graphed the OTUs identified in each microbiome using GraPhlAn (Asnicar, Weingart, Tickle, Huttenhower, & Segata, 2015). We used Qiime v1.7.0 to evaluate the alpha and beta diversity of the microbiomes, by constructing rank abundance curves to estimate species richness and evenness. To further measure alpha diversity, we calculated Good's coverage scores in mothur v1.39.5 (Wang, Garrity, Tiedje, & Cole, 2007), based on 10,000 iterations. We also measured the Shannon and Simpson alpha diversity indices, and calculated the chao1 and abundance‐based coverage estimator evenness metrics in R v3.2.2. To control for differences in sampling effort across the three environments, we rarefied each sample to 26,779 sequences before calculating diversity indices.

We then evaluated the beta diversity of the OTUs. To compare the composition of identified community members in different environments, we calculated a Bray–Curtis dissimilarity matrix based on the rarefied data (26,779 sequences per sample) and square‐root transformed the read abundance data (Beckers et al., 2017). We performed a principal component analysis (PCA) in R v3.2.2 to calculate the overall similarity in bacterial community structure across all environments. We then tested whether the OTUs in the different environments were significantly different using multiple response permutation procedure (Jammalamadaka, 2003) and the Spearman rank correlation in R v3.2.2.

MetaStat tests the correlation between the environment (Crucian carp gut, water, or mud) and the relative abundance of several bacterial phyla using hypothesis testing. We used the LEfSe (linear discriminant analysis [LDA] effect size) to statistically identify the most representative taxa in each environment. LEfSe combines statistical significance and biological correlation to identify the biological characteristics of taxa that are significantly more or less abundant.

## 3 RESULTS

### 3.1 Quality of sequencing analysis

We obtained 2,129,884 raw reads from the Illumina sequencing of the amplicon libraries. Average raw read length was 372 bp. After quality filtering, end trimming, and assigning reads to samples, 1,864,258 high‐quality reads remained (Supporting Information Table S1).

### 3.2 Taxonomic composition of the microbiomes

**Figure 1:**
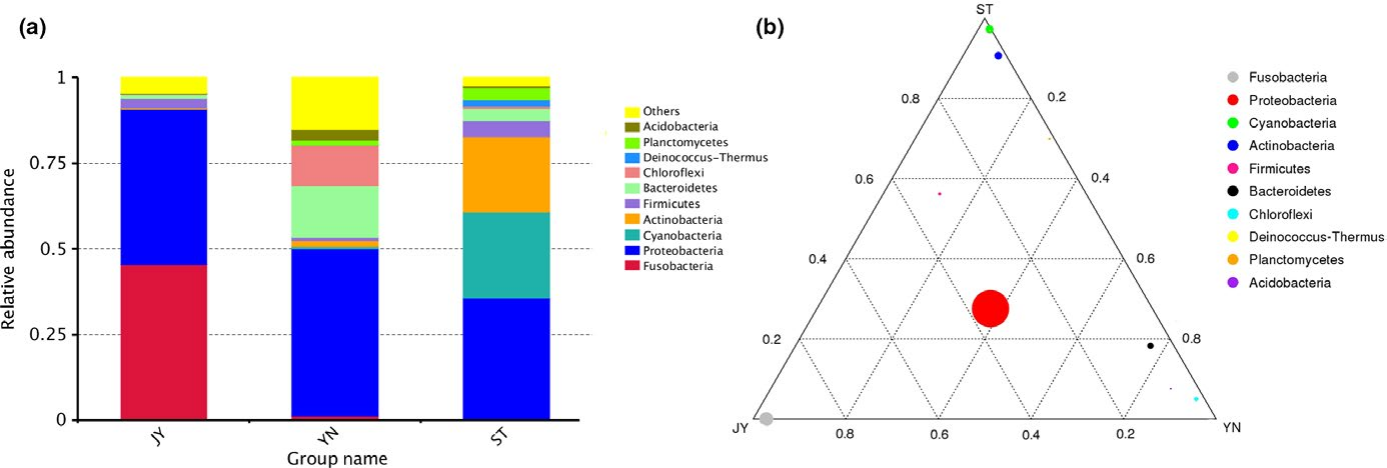
(a) Relative abundance of bacterial phyla in Crucian carp intestine, water, and mud. (b) Ternary phase diagram showing the relative abundance of various bacterial phyla. The three vertices represent the three environments, and each circle represents a different bacterial phylum. The size of each circle is proportional to relative abundance; the closer the circle is to the vertex, the higher the relative abundance of the group in that environment. JY: Crucian carp intestine; ST: water; YN: mud

**Figure 2:**
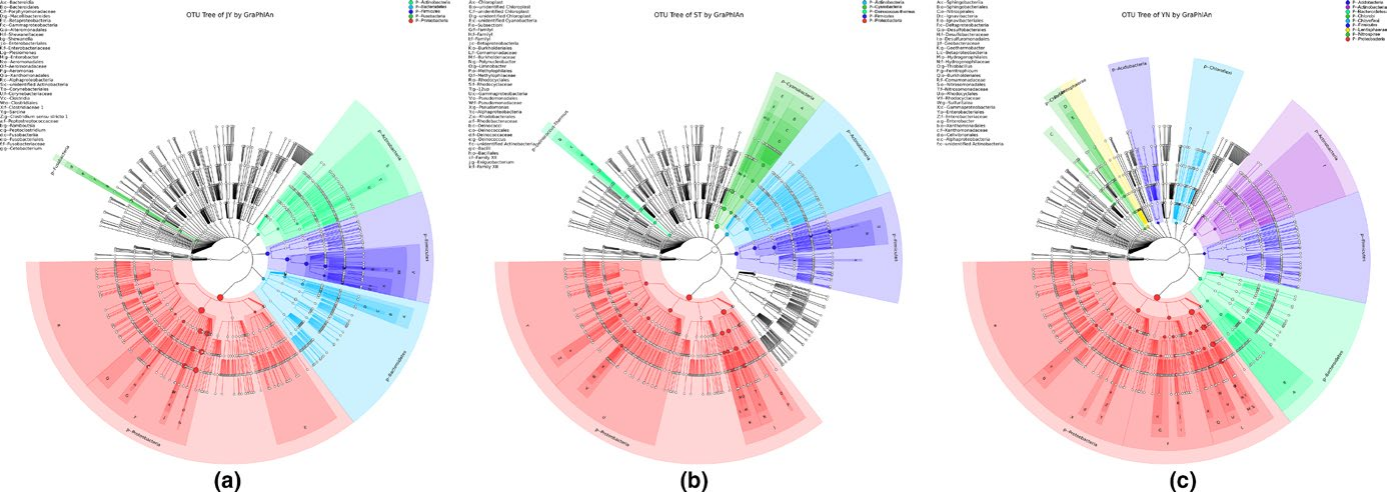
GraPhlAn phylogenies of the bacterial communities in each environment. The most abundant phyla in each environment are highlighted. (a) Crucian carp intestine; (b) water; (c) mud

The dominant bacterial phyla in the Crucian carp gut were Fusobacteria and Proteobacteria (≥90% of all bacterial phyla identified; Figure 1); in water, Proteobacteria, Cyanobacteria, and Actinobacteria (≥80% of all bacterial phyla identified; Figure 1); and in mud, Proteobacteria, Bacteroidetes, and Chloroflexi (≥70% of all bacterial phyla; Figure 1). The dominant bacterial families in the Crucian carp gut were Fusobacteriaceae, Enterobacteriaceae, and Aeromonadaceae (≥85% of all bacterial families identified). The dominant bacterial families in the water were Family I and XII, Sporichthyaceae, Comamonadaceae, Rhodocyslaceae, Burkholderiaceae, Methylophilaceae, Phycisphaeraceae, Acidimicrobiaceae, Rhodobacteraceae, Deinococcaceae, Moraxellaceae, Alcaligenaceae, and Planctomycetaceae (≥80% of all bacterial families identified). The dominant bacterial families in the mud were Hydrogenophilaceae, Anaerolineaceae, Draconibacteriaceae, WCHB‐69, Rhodocyclaceae, Comamonadaceae, Desulfarculaceae, Xanthomonadaceae, Aeromonadaceae, Flavobacteriaceae, BSV26, Nitrosomonadaceae, and Enterobacteriaceae (50% of all bacterial families identified). The dominant bacterial genera in the Crucian carp gut were *Cetobacterium*,* Aeromonas*, and *Plesiomonas* (≥85% of all bacterial genera identified). The dominant bacterial genera in the water were hgcI_clade, 12up, *Exiguobacterium*, unidentified_Chloroplast, CL500‐3, CL500‐29_marine_group, *Limnobacter*,* Polynucleobacter*,* Deinococcus*,* Pseudomonas*,* Synechococcus*, MWH‐UniP1_aquatic_group, *Acinetobacter*, and “Candidatus Planktophila.” The dominant genera in the mud were *Thiobacillus*,* Desulfatiglans*,* Aeromonas*,* Cetobacterium*,* Sulfuritalea*, and others (≥75% of all bacterial genera identified). To reduce the influence of bacterial outliers in the microbiome, we used mean values to identify dominant bacterial taxa. Variation in bacterial abundance within each habitat is shown in Supporting Information Figure S1. We use GraPhlAn with OTU Table to identify the OTUs in each environment (Figure 2).

### 3.3 Alpha rarefaction curves and alpha diversity

**Figure 3:**
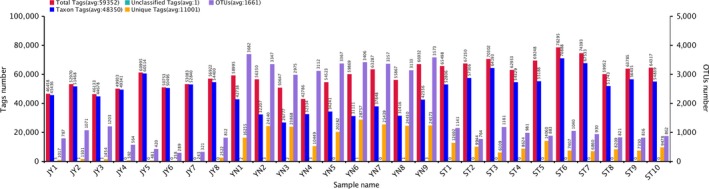
Average number of operational taxonomic units (OTUs) and unique OTUs in each environment

**Figure 4:**
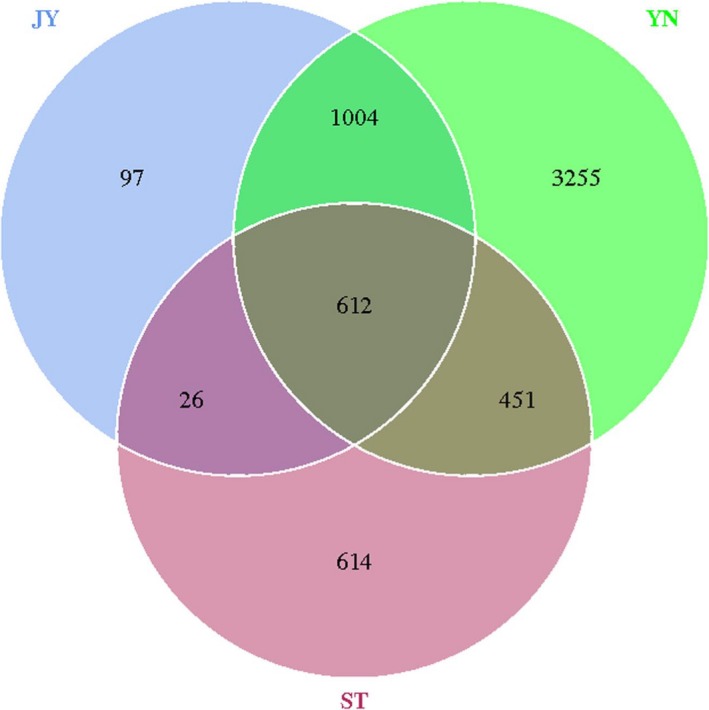
Venn diagram based on the number of operational taxonomic units (OTUs). JY: Crucian carp intestine; ST: water; YN: mud

Our alpha diversity metrics indicated that species richness and evenness was highest in the mud, and lowest in the fish gut (Table S2). We identified 1,739 OTUs in the Crucian carp gut, 1,703 in the water, and 5,322 in the mud (Figure 3). Most of the OTUs we identified occurred in multiple environments, with 612 OTUs occurring in all three environments (Figure 4). Nearly all the OTUs in the Crucian carp gut were also found in water and/or mud: 1,616 OTUs in both the fish gut and the mud, and 638 in both the fish gut and the water (Figure 4). Although we found OTUs unique to each environment, the Crucian carp gut had the fewest unique OTUs (97).

Good's coverage scores for all three environments were similarly high (range: 95.6%–99.6%) (Supporting Information Table S2), indicating that the sequencing depth we used was adequate for a reliable description of the bacterial microbiomes.

### 3.4 Beta diversity

**Figure 5:**
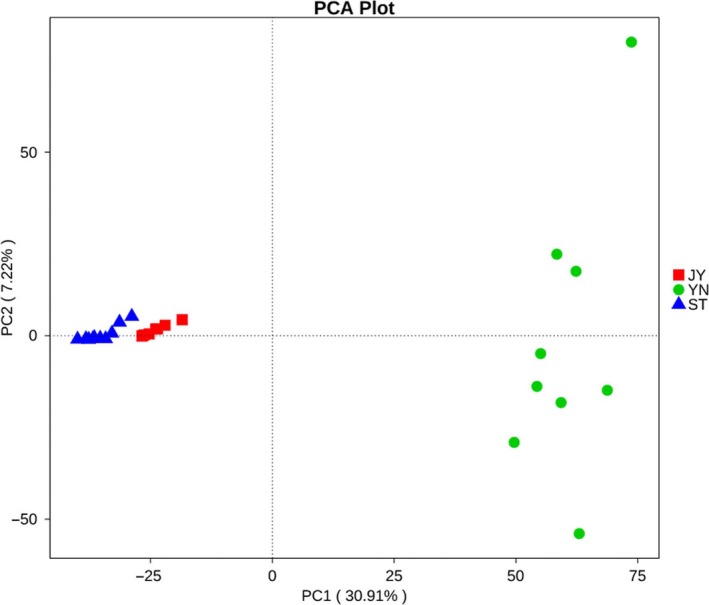
Principal components analysis (PCA) showing the clustering of bacterial operational taxonomic units (OTUs) by environment. PC1 explains 30.91% of the variance in the data; PC2 explains 7.22%. Green: mud; blue: water; red: Crucian carp intestine. JY: Crucian carp intestine; ST: water; YN: mud

Our PCA analysis indicated that the bacterial OTUs were strongly clustered by environment (Figure 5). PC1 and PC2 explained 30.91% and 7.22% of the total variance, respectively (Figure 5). The OTU compositions of the three environments were significantly different (*r* < 0.002; Supporting Information Table S3).

### 3.5 Variations in the bacterial microbiomes among environments

**Figure 6:**
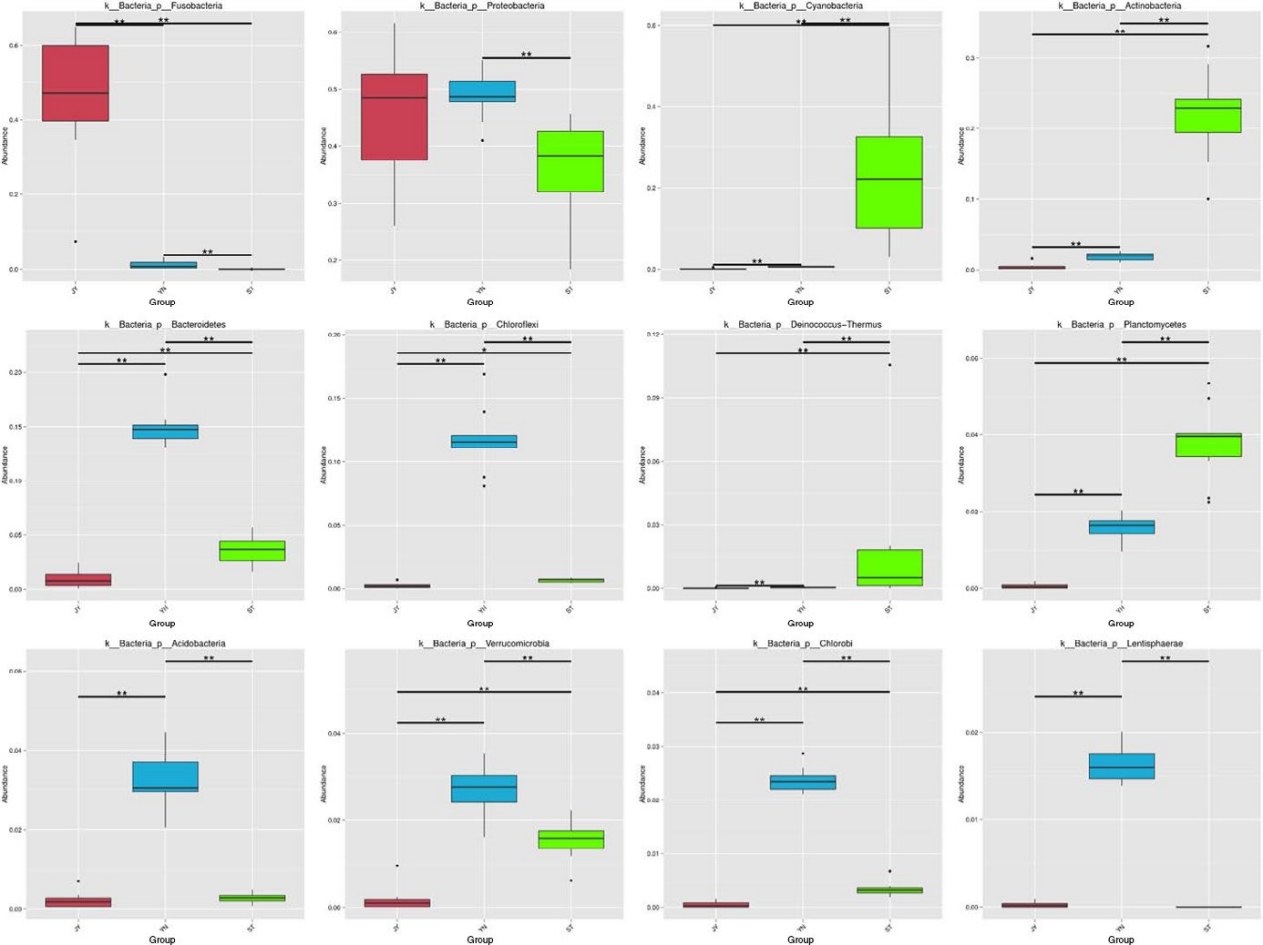
Differences in the abundance of the most abundant phyla in each environment. Bars shown mean abundance of all samples ± *SD*. Green bars: water (*n* = 10); blue bars: mud (*n* = 9); red bars: Crucian carp intestine (*n* = 6). *The difference between the pair of bars is statistically significant (*p* < 0.05)

**Figure 7:**
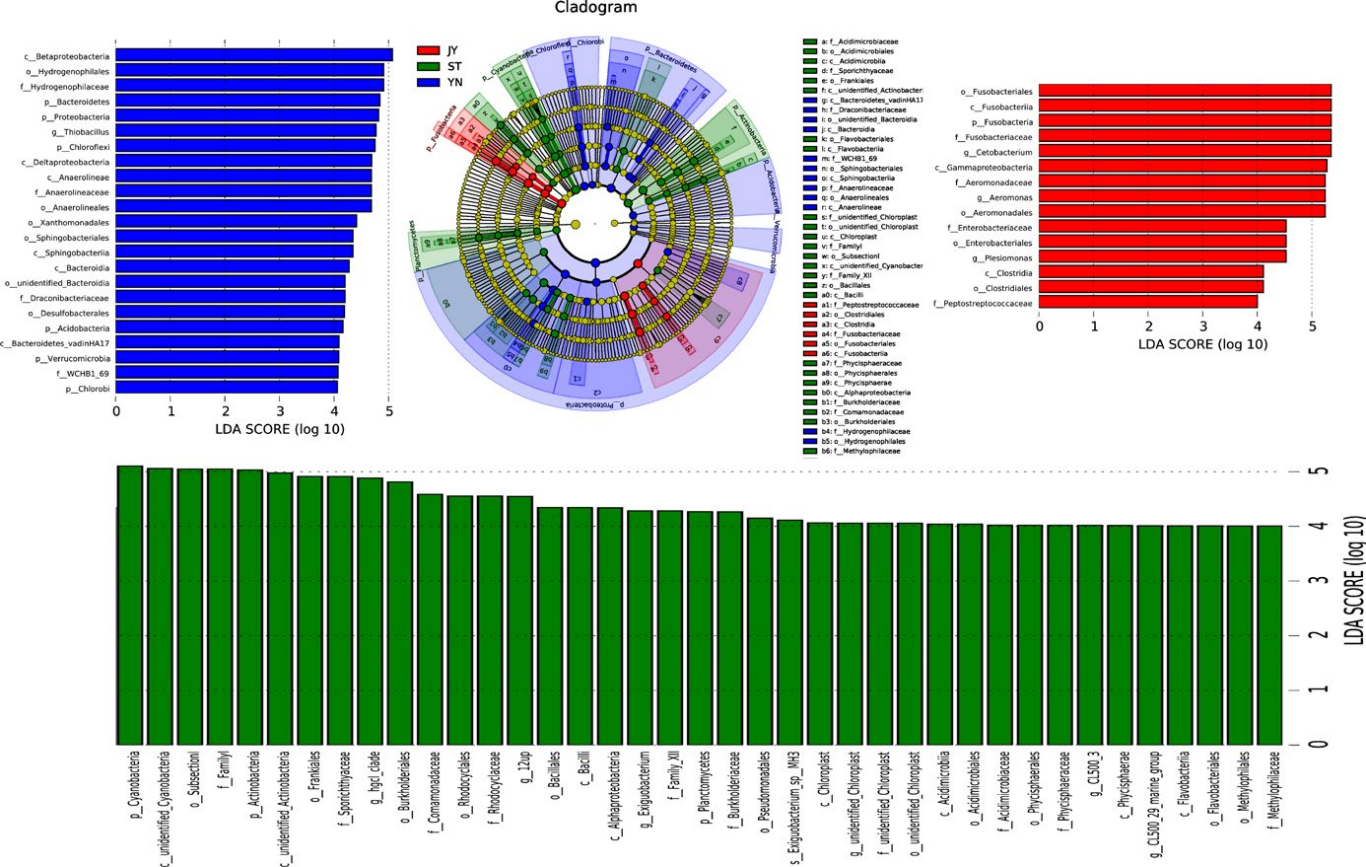
Differences in bacterial species abundance among the three environments identified using linear discriminant analysis (LDA) coupled with effect size (LEfSe). The LDA score histogram shows the biomarkers that differ significantly among groups. The degree of influence of each species is expressed by the length of the histogram. In the cladogram, the radiating circles demonstrate decreasing levels of classification (from phylum to genus). Each small circle represents an individual taxon, and the diameter of the circle is proportional to the relative abundance of that taxon. The species that do not differ significantly with respect to abundance are colored yellow. Biomarkers are colored based on group: red, green, and blue dots represent the core bacterial populations of the respective groups

Our MetaStat analysis indicated that the abundance of nearly all of the bacterial phyla varied significantly among the different environments (Figure 6). These phyla included Fusobacteria, Proteobacteria, Cyanobacteria, Actinobacteria, Bacteroidetes, Chloroflexi, Deinococcus‐Thermus, Planctomycetes, Acidobacteria, Verrucomicrobia, Chlorobi, and Lentisphaerae (Figure 7).

## 4 DISCUSSION

### 4.1 Microbiomes of the three environments

Our results were consistent with previous comparisons of the microbiotas of fish intestines and external environments (Sanchez, Weng, Riener, Schulze, & Linington, 2012; Schmidt, Smith, Melvin, & Amaral‐Zettler, 2015). The external environments of the Crucian carp used in this study were fresh water and mud. In fish, the external environment is the main factor affecting the intestinal microbiota. Previous studies have shown that the microbiotas of different environments have different microhabitats (Eichner, Erb, Timmis, & Wagner‐Döbler, 1999; Liu, Chan, & Fang, 2002; Pinto, Xi, & Raskin, 2012; Vaz‐Moreira, Nunes, & Manaia, 2014).

We found high alpha diversity in the mud, and moderate alpha diversity in the water. Nearly all of the OTUs identified in the Crucian carp gut were also found in water and/or mud. Indeed, ≥60% of the OTUs found in the Crucian carp gut overlapped with those found in the mud, but ≤1% of the OTUs found in the Crucian carp gut were also found in the water. This suggested that the mud may be the source of the microbiome found in the Crucian carp gut, and that the water was only a transferring medium. As can be indicated from the above data, Crucian carp can actively choose which bacteria to cultivate using the water medium from the mud.

Most of the bacteria found in the Crucian carp gut were Fusobacteria and Proteobacteria. Proteobacteria, Cyanobacteria, and Actinobacteria were most dominant in the fresh water, while Bacteroidetes and Chloroflexi were most dominant in the mud.

However, we found that the Fusobacteria is the dominant population in the Crucian carp gut while it was nondominant microbiota in all the mud samples and part of the water samples. The Crucian carp lived in the fresh water from its birth, so we predicted that the source of the Fusobacteria in the Crucian carp gut was the mud environment. The role of the freshwater is only a medium for transforming Fusobacteria to the fish gut.

### 4.2 How variable are the bacterial communities associated with different environments?

**Figure 8:**
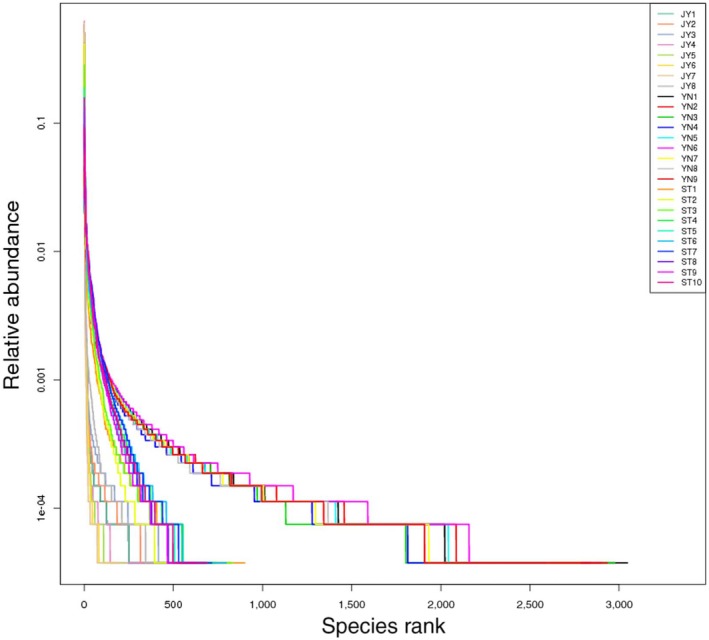
Rank abundance curves for each of the three environments tested. Each line represents an individual sample. Green lines: mud; blue lines: water; red lines: Crucian carp intestine

The rank abundance curves differed substantially among the three environments (Figure 8). Most of the OTUs we identified occur in all three environments. In the fish gut, only 5% of the OTUs we identified were unique. Therefore, our data suggested that most of Crucian carp intestinal microbiome comes from the external environment. Indeed, as the microhabitat of the intestine varies between aerobic and anaerobic, liquid and solid, it can host bacteria that typically inhabit both water and mud. These bacteria provide nutrition and energy to the Crucian carp host (Bäckhed et al., 2004). As Crucian carp are under extreme selective pressure to adapt to various environments (Matikainen & Vornanen, 1992), it may be that Fusobacteria and Proteobacteria have been retained by the fish only because they are required for basic vital activity and function. These structurally simple phyla dominate in the Crucian carp gut; it may be that their simple, mutable structure allows the bacteria to adapt more quickly to various aqueous environments.

In the fresh water samples, Cyanobacteria were one of the dominant bacterial phyla. Cyanobacteria are autotrophic prokaryotes that perform oxygenic photosynthesis in a similar manner to the higher plants (Chan et al., 2016). Cyanobacteria provide nutrition and energy to many aquatic organisms.

Two thirds of all the OTUs we identified were found in mud, including most of those found in the fish intestine. This suggested that most of the Crucian carp microbiome originated in the mud. It might therefore be possible to influence the composition of the fish microbiome by altering the bacterial composition of the mud.

Our study had a major limitation: we were unable to convincingly determine whether the bacteria shared between the mud and water samples originated from the mud or from the water. Future studies of environmental microbiota should consider the connections between different subsystems, as bacteria may be transported between these in various ways.

Further studies of the microbiota of aquatic environments will lead to a better understanding of fish health maintenance and assessment. With detailed knowledge of the interactions between the microbiotas of external and internal environments, aquaculture practices can be improved by, for example, altering the microbiotas of the external environment to promote beneficial bacterial growth in the fish intestine.

## CONFLICT OF INTEREST

The authors declare no conflicts of interest.
