# Supplementary figures and images for: Microorganism's adaptation of Crucian carp may closely relate to its living environments
Source: Microbiologyopen. 2018 Jun 6;8(3):e00650. doi: 10.1002/mbo3.650 (PMC6436428; doi:10.1002/mbo3.650)

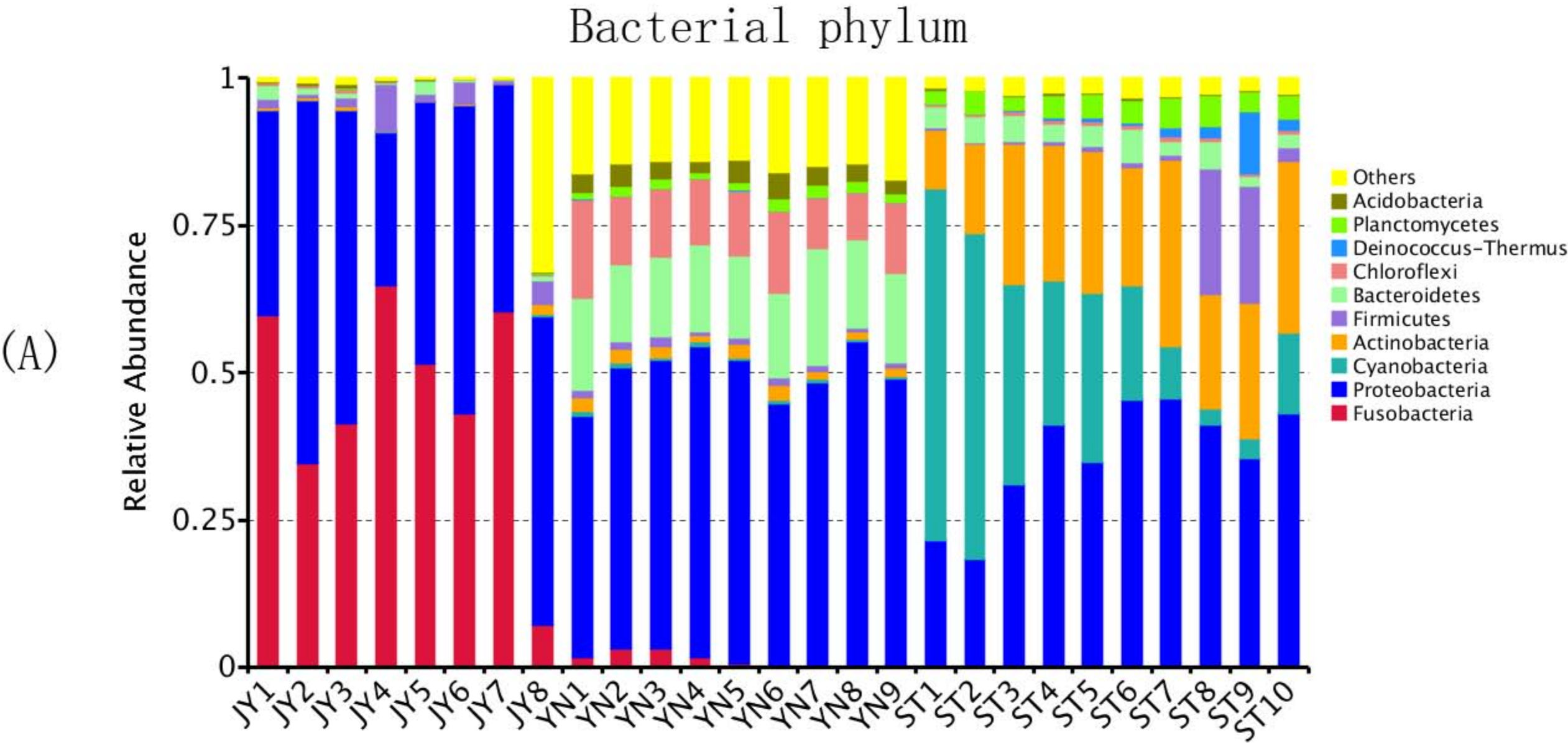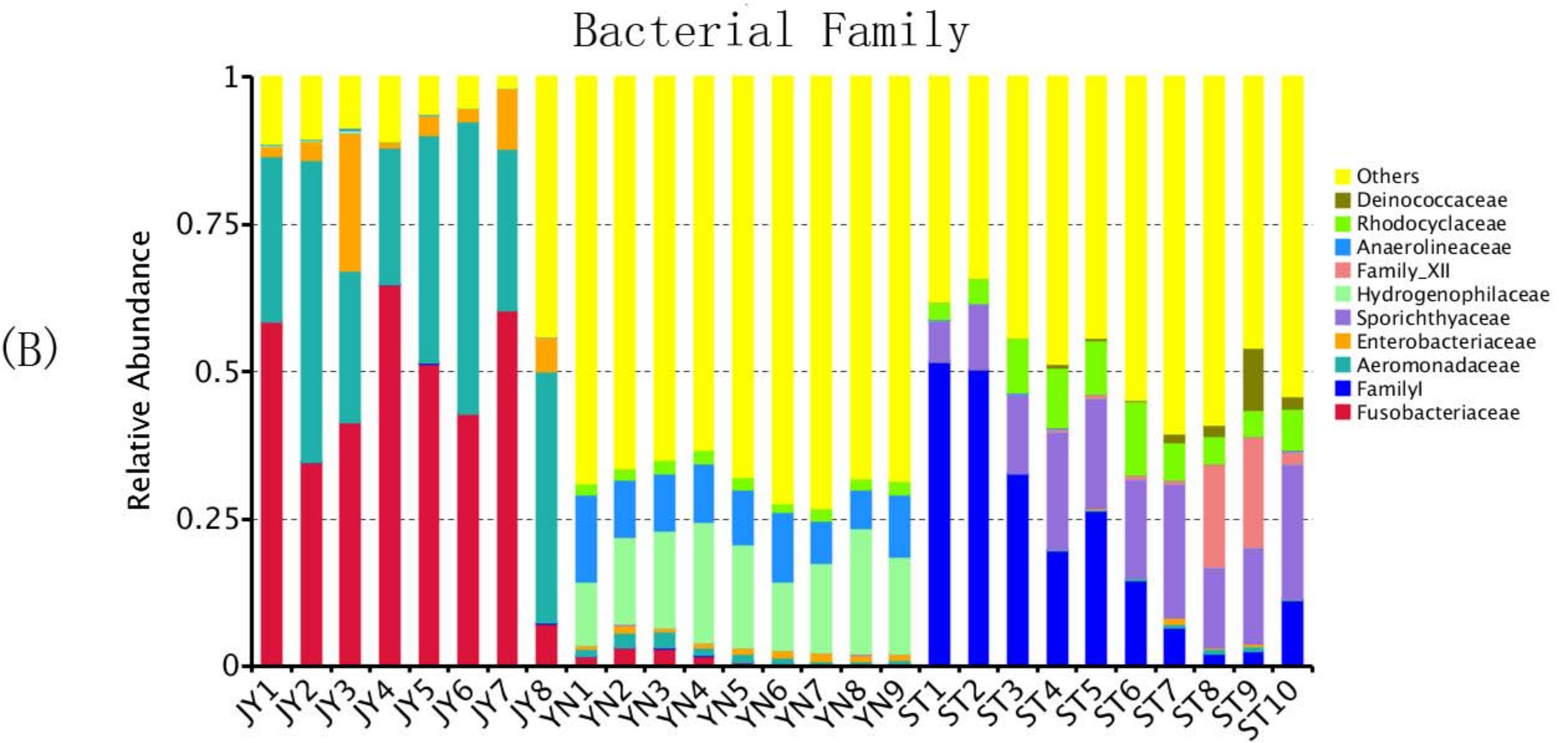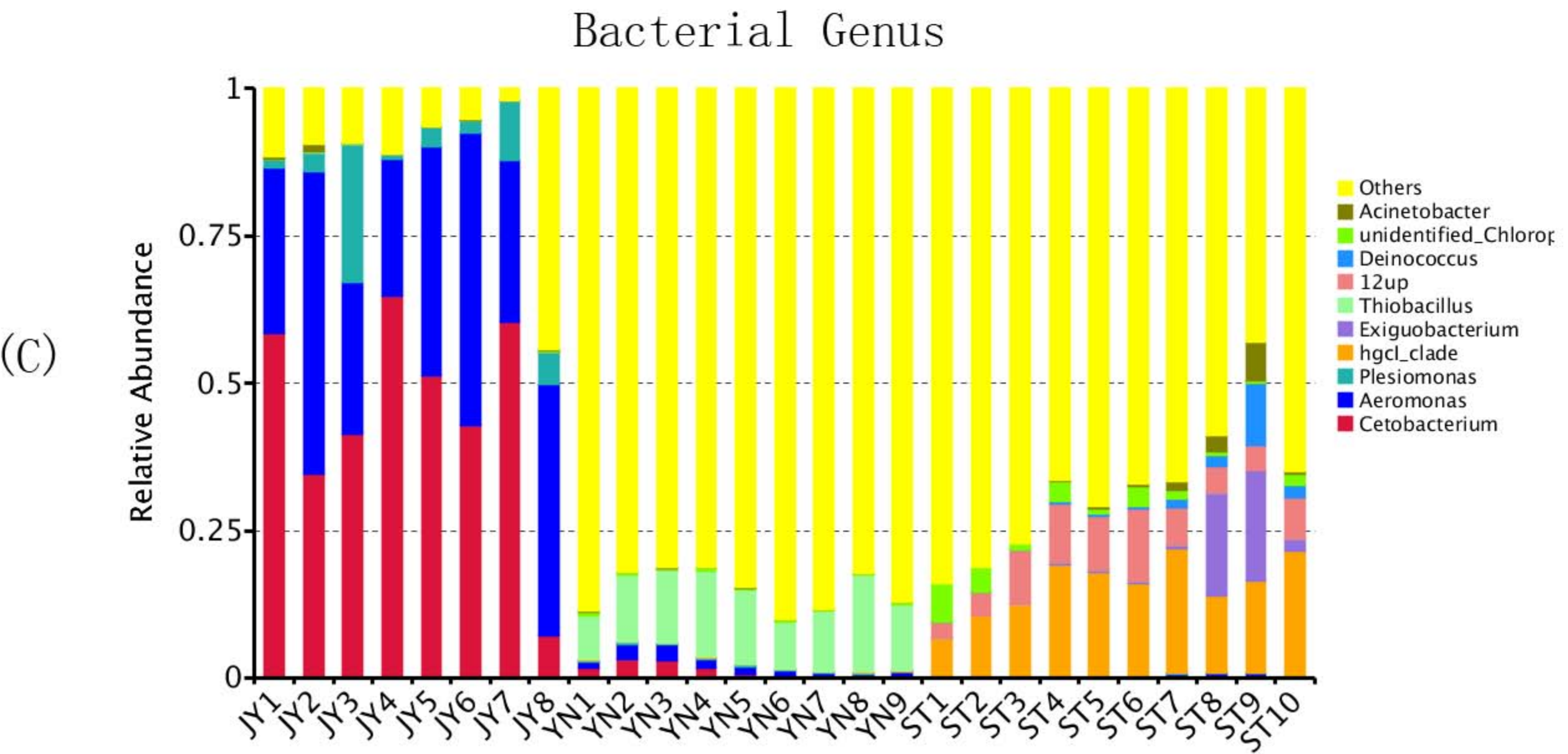

Supplement: Supplementary file 1 [file MBO3-8-e00650-s001.pdf]
