# Supplementary material for: Microorganism's adaptation of Crucian carp may closely relate to its living environments
Source: Microbiologyopen. 2018 Jun 6;8(3):e00650. doi: 10.1002/mbo3.650 (PMC6436428; doi:10.1002/mbo3.650)
Supplement: Supplementary file 2 [file MBO3-8-e00650-s002.docx]

**Supplementary Table 1. Sequencing quality metrics**

| Sample name | raw pe | raw tags | clean | Average length (bp) | Effect (%) |
| --- | --- | --- | --- | --- | --- |
| JY1 | 56,466 | 53,644 | 48,375 | 370 | 82.28 |
| JY2 | 65,615 | 62,336 | 56,142 | 371 | 80.73 |
| JY3 | 56,995 | 54,666 | 49,274 | 369 | 80.94 |
| JY4 | 69,703 | 66,766 | 60,242 | 370 | 71.64 |
| JY5 | 75,650 | 73,275 | 65,699 | 371 | 80.63 |
| JY6 | 62,705 | 59,702 | 53,345 | 371 | 80.94 |
| JY7 | 65,348 | 62,082 | 56,025 | 370 | 81.23 |
| JY8 | 76,900 | 74,884 | 67,444 | 370 | 74.02 |
| ST1 | 88,135 | 84,734 | 78,448 | 373 | 74.32 |
| ST10 | 91,944 | 87,907 | 82,308 | 373 | 69.95 |
| ST2 | 85,983 | 82,235 | 76,131 | 373 | 78.21 |
| ST3 | 84,884 | 81,676 | 75,864 | 373 | 82.82 |
| ST4 | 80,313 | 77,433 | 72,309 | 373 | 78.38 |
| ST5 | 91,704 | 87,919 | 82,462 | 373 | 75.51 |
| ST6 | 98,288 | 93,740 | 87,684 | 373 | 79.66 |
| ST7 | 94,944 | 90,741 | 85,022 | 373 | 78.35 |
| ST8 | 89,225 | 85,498 | 80,244 | 373 | 67.19 |
| ST9 | 91,178 | 87,408 | 82,133 | 373 | 69.96 |
| YN1 | 75,967 | 72,735 | 65,257 | 373 | 77.66 |
| YN2 | 79,580 | 76,965 | 68,939 | 373 | 70.81 |
| YN3 | 71,924 | 68,329 | 61,806 | 373 | 70.42 |
| YN4 | 55,891 | 53,098 | 48,018 | 373 | 76.55 |
| YN5 | 74,704 | 72,359 | 64,504 | 373 | 72.99 |
| YN6 | 83,734 | 80,373 | 71,973 | 373 | 71.5 |
| YN7 | 90,431 | 86,096 | 77,101 | 372 | 69.98 |
| YN8 | 81,308 | 77,896 | 69,837 | 373 | 68.71 |
| YN9 | 90,365 | 86,760 | 77,672 | 373 | 74.07 |
