# Supplementary material for: Microorganism's adaptation of Crucian carp may closely relate to its living environments
Source: Microbiologyopen. 2018 Jun 6;8(3):e00650. doi: 10.1002/mbo3.650 (PMC6436428; doi:10.1002/mbo3.650)
Supplement: Supplementary file 3 [file MBO3-8-e00650-s003.docx]

**Supplementary Table 2** Comparison of phylotype coverage and diversity estimates for the 16S ribosomal RNA gene libraries for the microbiome samples from the three environments, at 3% dissimilarity.

| Sample name | OTUs | Shannon's Diversity Index (H) | Simpson's Diversity Index (SDI) | Chao1 | ACE | Good's  coverage |
| --- | --- | --- | --- | --- | --- | --- |
| JY1 | 536 | 2.174 | 0.583 | 866.976 | 965.982 | 0.989 |
| JY2 | 724 | 2.39 | 0.625 | 1356.091 | 1404.163 | 0.985 |
| JY3 | 822 | 2.807 | 0.721 | 1299.994 | 1399.853 | 0.985 |
| JY4 | 373 | 2.01 | 0.559 | 748.043 | 819.593 | 0.991 |
| JY5 | 233 | 1.888 | 0.602 | 382.529 | 435.256 | 0.995 |
| JY6 | 181 | 1.713 | 0.582 | 312.108 | 384.276 | 0.996 |
| JY7 | 201 | 1.609 | 0.558 | 399.244 | 554.109 | 0.995 |
| JY8 | 579 | 2.921 | 0.715 | 784.187 | 855.806 | 0.991 |
| YN1 | 3046 | 9.098 | 0.989 | 3920.417 | 4050.751 | 0.962 |
| YN2 | 2839 | 8.843 | 0.982 | 3289.124 | 3428.465 | 0.972 |
| YN3 | 2975 | 8.625 | 0.979 | 3993.11 | 4422.612 | 0.956 |
| YN4 | 2589 | 8.424 | 0.974 | 3147.52 | 3248.099 | 0.971 |
| YN5 | 2774 | 8.813 | 0.98 | 3195.997 | 3340.089 | 0.973 |
| YN6 | 2899 | 9.247 | 0.99 | 3380.845 | 3431.818 | 0.972 |
| YN7 | 2782 | 8.77 | 0.984 | 3349.335 | 3547.149 | 0.968 |
| YN8 | 2608 | 8.361 | 0.967 | 3055.156 | 3130.72 | 0.974 |
| YN9 | 2933 | 8.832 | 0.983 | 3860.208 | 3967.825 | 0.962 |
| ST1 | 900 | 4.805 | 0.828 | 1315.932 | 1325.614 | 0.987 |
| ST2 | 561 | 4.632 | 0.818 | 685.874 | 738.34 | 0.994 |
| ST3 | 832 | 5.459 | 0.903 | 1056.833 | 1141.692 | 0.989 |
| ST4 | 741 | 6.262 | 0.959 | 885.925 | 915.442 | 0.993 |
| ST5 | 679 | 5.96 | 0.944 | 811.839 | 836.52 | 0.994 |
| ST6 | 796 | 6.471 | 0.964 | 1127.154 | 1063.395 | 0.991 |
| ST7 | 714 | 6.787 | 0.977 | 901.033 | 888.049 | 0.993 |
| ST8 | 684 | 6.535 | 0.972 | 924.191 | 941.232 | 0.992 |
| ST9 | 629 | 6.137 | 0.957 | 766.531 | 780.877 | 0.994 |
| ST10 | 688 | 6.394 | 0.969 | 865.892 | 878.22 | 0.993 |
