# Supplementary material for: Microorganism's adaptation of Crucian carp may closely relate to its living environments
Source: Microbiologyopen. 2018 Jun 6;8(3):e00650. doi: 10.1002/mbo3.650 (PMC6436428; doi:10.1002/mbo3.650)
Supplement: Supplementary file 4 [file MBO3-8-e00650-s004.docx]

**Supplementary Table 3.** MRPP statistical analysis using Spearman's rank correlation.

| Groups compared | A | observed-delta | expected-delta | Significance |
| --- | --- | --- | --- | --- |
| JY-YN | 0.5483 | 0.2846 | 0.6301 | 0.002 |
| ST-YN | 0.5035 | 0.3269 | 0.6585 | 0.001 |
| JY-ST | 0.4773 | 0.3613 | 0.6913 | 0.001 |
